# Ligand dependent restoration of human TLR3 signaling and death in p53 mutant cells

**DOI:** 10.18632/oncotarget.11210

**Published:** 2016-08-11

**Authors:** Daniel Menendez, Julie M. Lowe, Joyce Snipe, Michael A. Resnick

**Affiliations:** ^1^ Genome Integrity & Structural Biology Laboratory, Inflammation Disease Laboratory, National Institute of Environmental Health Sciences, NIH, Research Triangle Park, NC 27709, USA; ^2^ Immunity, Inflammation Disease Laboratory, National Institute of Environmental Health Sciences, NIH, Research Triangle Park, NC 27709, USA

**Keywords:** apoptosis, poly (I:C), innate immunity, mutant p53, cancer therapy

## Abstract

Diversity within the p53 transcriptional network can arise from a matrix of changes that include target response element sequences and p53 expression level variations. We previously found that wild type p53 (WT p53) can regulate expression of most innate immune-related Toll-like-receptor genes (*TLRs*) in human cells, thereby affecting immune responses. Since many tumor-associated p53 mutants exhibit change-of-spectrum transactivation from various p53 targets, we examined the ability of twenty-five p53 mutants to activate endogenous expression of the *TLR* gene family in p53 null human cancer cell lines following transfection with p53 mutant expression vectors. While many mutants retained the ability to drive *TLR* expression at WT levels, others exhibited null, limited, or change-of-spectrum transactivation of *TLR* genes. Using TLR3 signaling as a model, we show that some cancer-associated p53 mutants amplify cytokine, chemokine and apoptotic responses after stimulation by the cognate ligand poly(I:C). Furthermore, restoration of WT p53 activity for loss-of-function p53 mutants by the p53 reactivating drug RITA restored p53 regulation of *TLR3* gene expression and enhanced DNA damage-induced apoptosis via TLR3 signaling. Overall, our findings have many implications for understanding the impact of WT and mutant p53 in immunological responses and cancer therapy.

## INTRODUCTION

The tumor suppressor p53 is a sequence-dependent transcription factor that is critical for several signaling events in response to a variety of stress signals. Once activated, p53 orchestrates expression of many genes that regulate transient cell cycle arrest, senescence, DNA repair and cell death, which all aid in tumor suppression [1]. The importance of p53 as a tumor suppressor is demonstrated by the fact that *TP53* gene mutations are the most frequent somatic events in cancers [2, 3]. Most cancer-associated p53 mutations are missense and mostly located in the DNA binding domain of the protein, affecting its transcription factor activities. Among the different mutations that have been associated with cancer, approximately one-third retain transactivation capability [4–7]. Many of these can result in a change-of-spectrum transactivation of various p53 targets, thereby altering subsequent cellular responses such as DNA repair, genome stability and programmed cell death that facilitates tumorigenesis [4, 6, 8]. Since many tumors express mutant p53, there is a large effort to identify small molecules, such as RITA [9], that restore p53 tumor suppressor functions in tumor cells harboring mutant p53.

Genome-wide analyses using chromatin immunoprecipitation (ChIP) followed by next generation DNA sequencing and expression profiling have increased the list of validated p53-regulated genes beyond just regulation of cell cycle and cell fate that respond to cellular and genotoxic stresses. Several additional cellular processes that are also important to p53 tumor suppressor activities [10–12] have been revealed including genes involved in stem cell maintenance, restriction of invasion and metastasis, metabolism, autophagy and communication within the tumor microenvironment including immune responses [13]. Recent studies have emphasized the role of p53 in influencing and modulating the human immune system against tumors [14, 15]. For example, DNA damage can trigger p53 responses that help orchestrate clearance of damaged cells via the innate immune system [16, 17].

The Toll-like receptors (TLRs) play a key role in host defense against invading pathogens, mediating immediate and early host defense responses as well as orchestrating adaptive immune responses. TLRs are membrane glycoproteins that function as primary sensors of pathogen-associated molecular patterns (PAMPS) from viruses, bacteria, fungi and parasites. The human TLR gene family consists of ten genes, TLR1 to TLR10, and each TLR recognizes distinct PAMPs. For example, TLR4 on the cell surface detects gram-negative bacterial lipopolysaccharide (LPS), and TLR3 located in endosome vesicles recognizes viral dsRNA [18]. After ligand activation, TLRs orchestrate downstream signaling pathways involving adaptor proteins, protein kinases and effector transcription factors that ultimately induce expression of pro-inflammatory mediators including cytokines, chemokines and interferons [19, 20]. TLRs are required not only for the organization of innate responses to pathogens but also for optimal activation of the immune system against cancer [21]. Since TLRs also can modulate adaptive immune responses, there has been an emphasis on TLR-based therapeutic approaches that enhance the efficiency of anticancer immunotherapies [22]. However, there have been conflicting reports concerning the pro- or antitumoral role of several TLRs [18].

Recently, our lab and others identified *TLR* human gene family members as p53 targets and responsive to chromosomal stress. We also established that this responsiveness is not available in rodents. Exposure of various human immune-related primary cells as well as cancer-derived cells to common anticancer agents led to p53-dependent modulation of most *TLR* genes resulting in a synergistic increase of downstream responses to cognate ligands for the *TLR2, TLR3* and *TLR5* [23–26]. Previously, we described a small number of tumor-associated p53 mutants that when transiently expressed in human cancer cells dramatically influenced the expression of some TLR genes [25]. However, the impact of those p53 mutations on downstream TLR signaling was not elucidated, nor was the effect of stress conditions.

Based on our previous results, we anticipated that p53 mutations might alter the p53 responsiveness of immune pathways so that a combination of immune ligand along with chemotherapeutically induced p53 might alter inflammatory and immune type responses against tumors. Here, we have addressed the consequences of p53 mutants identified in human cancers on challenges to the TLR component of the immune system. We investigated the impact of twenty-five cancer-associated p53 mutants on expression of the *TLR* gene family. Using the TLR3 pathway as proof-of-principle, we examined the impact of the p53 mutants on downstream immune signaling in response to a TLR3 cognate ligand and chemotherapeutic drugs. Since TLR3 along with its downstream signaling responses have anti-cancer properties including immune-mediated tumor growth suppression and a direct apoptotic effect on TLR3 expressing cancer cells [27–30], TLR3 is currently being investigated for therapeutic interventions in cancer treatment [21, 31]. We found that p53 mutants that retain the ability to transactivate *TLR3*, can be used together with the TLR3 agonist polyinosinic-polycytidylic acid (poly(I:C) to alter immune responses mediated by this receptor and subsequently increase TLR3-mediated apoptosis. Moreover, we demonstrate that the functional recovery of p53 activity with the reactivating drug RITA can restore p53 responsiveness of *TLR3* gene expression including enhanced DNA damage-induced apoptosis via TLR3 poly(I:C) signaling.

## RESULTS

### p53 mutants can differentially modify expression of the TLR gene family

**Figure 1:**
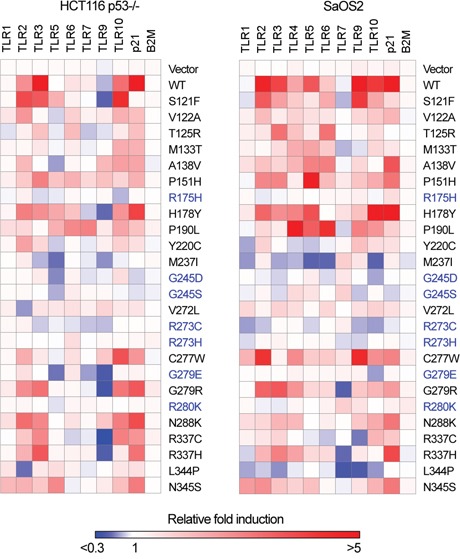
Differences in TLR gene expression profiles between WT and p53 mutants Heat map for *TLR* gene expression profiles in HCT116 p53−/− and SaOS2 cells transfected with p53 mutants. Expression of *TLRs* twenty-four h after transfection in three replicates was assessed by qPCR. Presented in the heat map is the relative-fold change in mRNA in transfected vs the parental nontransfected cells. The cancer hotspot loss-of-function group of mutants are depicted in blue. Fold-changes values are available in the Supplementary Table S2.

Previously, we had established that WT p53 can modify responsiveness of most TLRs in several human cancer cell lines in a tissue-dependent manner [25]. Since p53 mutants vary in their ability to induce transactivation from p53 targets, we assessed the potential for p53 mutant proteins expressed from vectors to modulate expression of endogenous *TLR* genes in two human cancer cell lines that are p53 null. A total of 25 different p53 mutants including 21 that are cancer-associated were evaluated (Supplementary Table S1). The mRNA levels expressed from the various *TLR*s were evaluated approximately 24 h after transfection, as described in the heat map in Figure 1.

Transient expression of WT p53 in SaOS2 osteosarcoma cells resulted in induction of 7 of the 10 *TLR* genes, while in HCT116 colon cells expression was significantly increased for only 4 genes. The responses of the established p53 target gene *p21(CDKN1A)* and the nontarget beta 2 microglobulin (*B2M*) gene were included as controls. Consistent with our previous findings, *TLR8* mRNA was not detected in either cell type while *TLR4* was only expressed in SaOS2 cells [25].

When compared to the expression profile induced by WT p53, several p53 mutants (S121F, P151H, H178Y, G279R) retain the ability to induce or repress at similar levels the expression of at least one *TLR* gene, while another group that included T125R, M133T, A138V, P190L, V272L, C277W and R337H exhibited a limited transactivation potential as well as change-of-spectrum of *TLR* genes expressed. Cell line-specific expression patterns were also observed, as for the case of *TLR9.* In HCT116, expression of WT p53 and several mutants resulted in a reduced *TLR9* expression while in SaOS2 there was increased expression, suggesting the involvement of cell specific co-regulatory factors. The loss-of-function group of mutants R175H, G245D, G245S, R273C and G279E (depicted in blue in the Figure 1) did not induce any *TLR* genes in either cell line, excluding the possibility of gain-of-function activity [32] for these mutants towards some genes. For *p21* expression, the mutants showed various transactivation profiles, supporting the diversity in transactivation activities by p53 mutants. Thus, several tumor-associated p53 mutants that retain transcriptional activities can modulate the innate immune response.

### p53 mutants can enhance TLR3 induced immune and apoptotic responses

We hypothesized that some of the p53 mutants could also influence the downstream signaling mediated by TLRs in response to their cognate ligands, as described previously for WT p53 [24–26]. We identified a group of p53 mutants that changed the pattern of *TLR* expressed genes relative to WT p53 in at least one of the two cell lines evaluated. For example, a group of p53 mutants that included M133T, A138V, P190L and C277W have decreased ability to drive the expression of *TLR3*, while the activity of the other p53 variants towards *TLR3* such as P151H, H178Y, G279R and R337H was similar to WT p53 (Figure 1). Our subsequent studies reported here focus on the p53/TLR3 interactions as a model for other possible studies on p53/TLR relationships.

**Figure 2:**
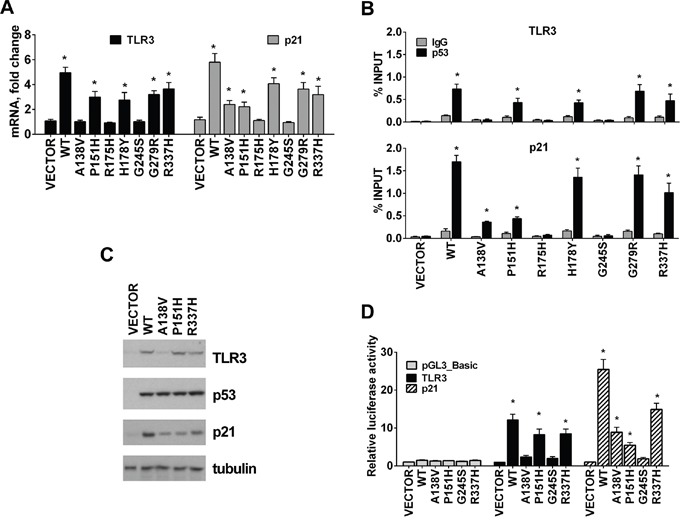
*TLR3* expression varies between tumor associated p53 mutants **A.** Several p53 tumor associated mutants change the profile of *TLR3* and *p21* expression determined by qPCR when expressed in p53 null HCT116 cells. **B.** Changes in *TLR3* expression are associated with differential p53 mutant binding in the promoter region of this gene. Similar to WT p53, the p53 mutants P151H and R337H but not A138V bound the TLR3 p53 RE. Differential binding by these mutants was also observed for the p53 RE associated with p21. IgG serves as a negative control. **C.** TLR3, p53 and p21 protein levels in cells transfected with WT and p53 mutants. Tubulin detection was used as loading control. **D.** HCT116 p53−/− cells were transfected with pGL3_Basic reporter containing the *TLR3* or *p21* promoter and co-transfected with pCMV empty vector (VECTOR) or p53 expression plasmid; luciferase activity was assessed 24 h after transfection. Values were normalized to Renilla luciferase activity. Relative luciferase against “VECTOR” condition is presented. Presented are means and standard deviations from triplicate determinations from 3 independent experiments. * p<0.001.

As shown in Figure 2A-2B, changes in *TLR3* gene expression (Figure 2A) correlate well with differential p53 binding in the promoter region of this gene based on ChIP assays (Figure 2B) following expression of some of the above p53 mutants. Similar to WT p53, the p53 mutants P151H, H178Y, G279R and R337H, but not A138V, bound the *TLR3* p53 response element located in the promoter region (Figure 2B) and enhanced *TLR3* mRNA levels (Figure 2A). Binding by these mutant proteins was also observed at the p53 RE associated with p21 although there were relative differences, as for A138V and H178Y, suggesting that p53 mutants can have different specificities for p53 binding sequences. No binding was observed at the promoter region of the negative control GAPDH (Supplementary Figure S1). The increase in *TLR3* and *p21* mRNA levels induced by these p53 mutants corresponded to increases in these proteins (Figure 2C). The loss-of-function mutants R175H and G245S, which are unable to interact with canonical p53 binding sequences or induce *TLR3* expression, were used as negative controls in these experiments and showed no binding to the *TLR3* promoter region or increased *TLR3* mRNA levels, as expected.

We also confirmed that P151H and R337H positively regulated *TLR3* expression in a luciferase reporter assay using the promoter region of TLR3, which contains a validated binding site for p53 [23]. Like WT p53, the expression in HCT116 p53−/− cells of P151H and R337H mutants, but not A138V, induced reporter gene expression, as shown in Figure 2D. The *p21* reporter responses were also consistent with altered binding and transactivation spectra of the A138V, P151H and R337H mutants. Taken together, p53 mutants proficient for binding to the *TLR3* promoter can activate *TLR3* transcription and protein expression in a manner that can be unique to each mutant and not a simple overall modulation of the WT p53 response.

**Figure 3:**
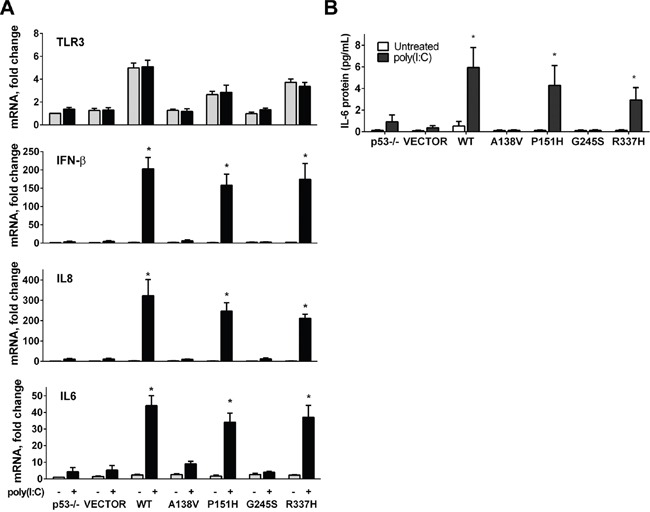
p53 mutants enhance TLR3 induced immune responses **A.** mRNA expression of *TLR3, IL-8, IFN-β* and *IL-6* was analyzed in HCT116 p53−/− cells that were first transfected with WT or mutant p53 for 24 h and then exposed (black bars) or not (gray bars) for 3 h to the TLR3 ligand poly(I:C) (5μg/mL). **B.** Secretion of IL-6 after ligand treatment in cells transfected with WT or mutant p53 mutant as measured by ELISA. Data presented are means and standard deviations from triplicate determinations from 3 independent experiments. * p<0.001.

TLR3 can directly trigger apoptosis in human cancer cells in response to the double-strand RNA synthetic agonist polyinosinic-polycytidylic acid (poly(I:C)) [28, 29], which is a mimic for viral double-stranded RNA that triggers an innate immune response following viral infection. In response to poly(I:C), TLR3 induces a type I interferon (IFN) along with inflammatory cytokine/chemokines. We reasoned that the combination of poly(I:C) and TLR3 downstream signaling would provide a model system for directly addressing the influence that p53 mutants might have on potential TLR signaling. We examined the effect of the p53 mutants on poly(I:C) stimulation of expression of several downstream inflammatory genes in HCT116 p53^−/−^ cells transfected with the change-of-spectrum p53 mutants. Although *TLR3* expression could be increased by WT p53 and some of the mutants (P151H and R337H), the levels were not further increased by poly(I:C) (Figure 3A). In HCT116 p53 null cells the basal levels of TLR3 are 50% less than in the HCT116 p53+/+ cells [23]. Transient transfection with WT and plasmids expressing mutant p53 had no apparent influence on the expression of the *IL-8 (CXCL8), IFN-β (IFNB1)* and *IL-6*, nor did poly(I:C) (3h) on parental p53^−/−^ cells or those transfected with the empty vector. However, like the WT p53, some of the p53 mutants could dramatically increase the poly(I:C) induced levels of *IL-8, IFN-β* and *IL-6* mRNA. In cells expressing WT p53 and mutants P151H and R377H a significant induction of *IL-8, IFN-β* and *IL-6* mRNA levels was observed after poly(I:C) treatment, whereas only a slight induction of cytokines resulted with A138V and G245S mutants (Figure 3A). Treatment with poly(I:C) had no impact on WT or p53 mutant mediated expression of the p53 target genes *p21* and *PUMA (BBC3)* (Supplementary Figure S2). The stimulatory impact that *TLR3* expression driven by p53 mutants has on the TLR3 response to poly(I:C) was also observed for secretion of the proapoptotic cytokine IL-6 as determined by ELISA (Figure 3B). Overall, our results show that some p53 mutants can synergistically enhance TLR3 induced immune responses.

**Figure 4:**
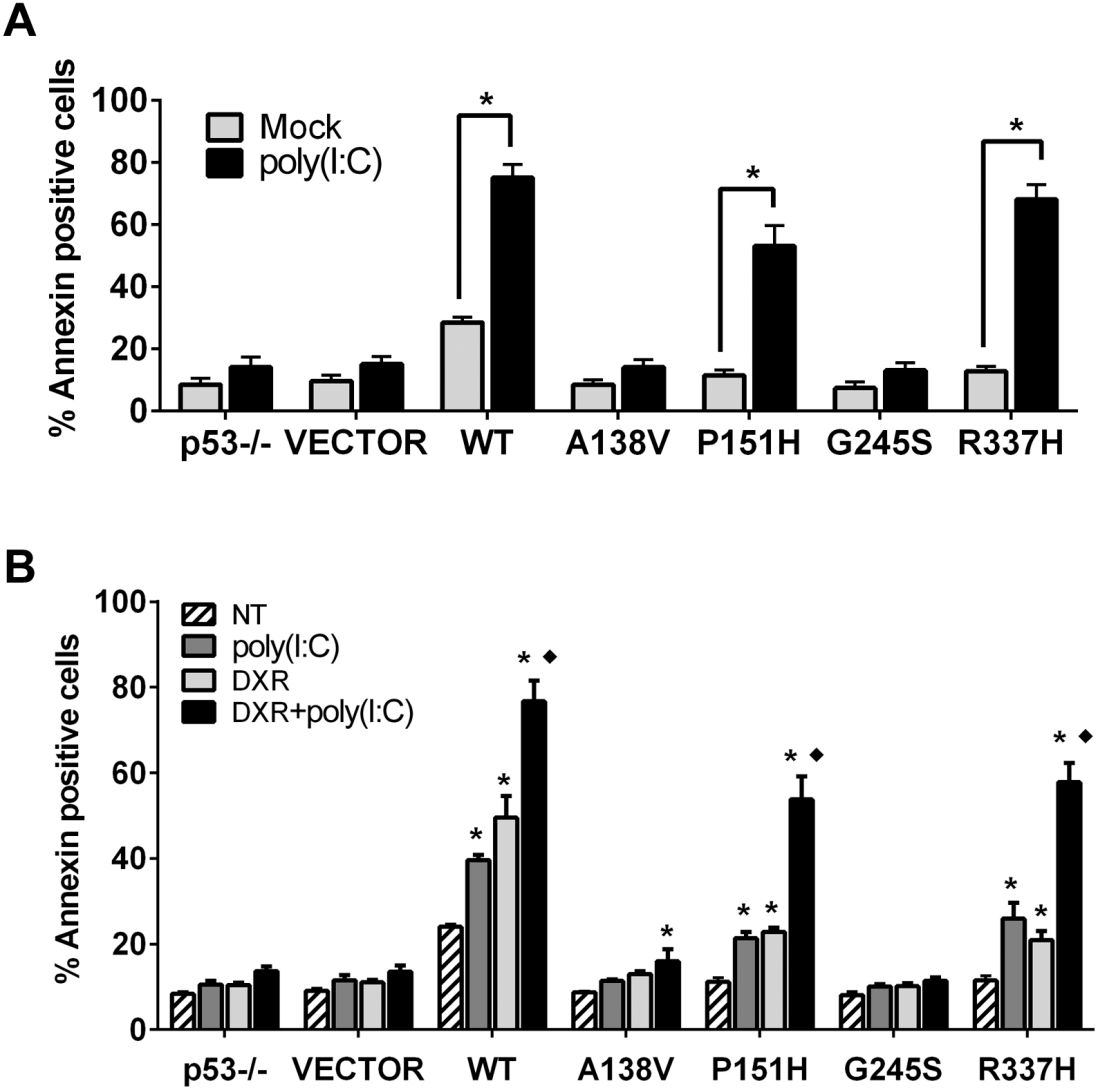
p53 mutants enhance TLR3 induced apoptotic responses **A.** Modulation of TLR3 signaling by p53 mutants induces apoptosis. HCT116 p53−/− cells were transfected with WT and mutant p53 vectors. After 24 h cells were incubated with the TLR3 ligand poly(I:C) (5 μg/mL) and apoptosis was analyzed 24 h later with the Annexin V/PI assay. The results correspond to the Annexin positive cells as a marker of apoptosis. **B.** DXR treatment enhances poly(I:C) induced apoptosis in a p53 mutant-dependent manner. Twenty-four h post transfection, p53 mutant-expressing cells were co-treated with DXR 0.5 μM and poly(I:C). 24 h later apoptosis was evaluated as described. Presented are means and standard deviations from triplicate determinations from 3 independent experiments. * p<0.001 relative to NT condition, ♦ <0.001 relative to DXR and poly(I:C) treatment alone.

Activation of TLR3 signaling by its agonists can induce apoptosis in human cancer cells [29, 33]. Based on the above results, we asked whether the p53 mutants that increased TLR3 levels and downstream inflammatory responses also enhanced the poly(I:C) apoptotic response. As shown in Figure 4A, 24h exposure to poly(I:C) significantly increased the incidence of apoptosis in P151H and R337H expressing cells, as indicated by Annexin V staining, similar to WT p53. There was no increase for the A138V and G245S transfected cells. The impact of poly(I:C) on cell survival in p53 mutant expressing cells was also confirmed with a MTT (3-(4,5-dimethylthiazol-2-yl)-2,5-diphenyltetrazolium bromide) based assay. The levels of cell death induced by WT p53, P151H and R337H in the presence of poly(I:C) were comparable to those detected in the isogenic HCT116 p53+/+ cells treated with poly(I:C) (Supplementary Figure S3A). Apoptosis in the p53 mutant expressing cells in response to poly(I:C) was dependent on caspase activity since the pan-caspase inhibitor z-VAD-fmk prevented the appearance of apoptotic cells (Supplementary Figure S3B). Furthermore, the poly(I:C) induced cell death in mutant p53 cells was significantly enhanced by co-treatment with the anticancer drug DXR (Figure 4B) when compared with either treatment alone. Overall, these data support the view that altering the TLR3 pathway, including increased TLR3 levels and downstream ligand induced activation, can potentially be exploited for cancer treatments utilizing existing mutants or WT p53 in combination with chemotherapeutic drugs.

### Functional reactivation of p53 mutants modulates TLR3 induced downstream signaling

Although p53 change-of-spectrum mutants can impact TLR expression and signaling, their frequency in tumors is low when compared with classical p53 hotspot mutants, which have lost the ability to interact with p53 binding sequences. As expected, these loss-of-function hotspot mutants do not influence *TLR* gene family expression (Figures 1 and 2A). We, therefore, investigated whether functional reactivation of endogenous loss-of-function mutants can restore p53-induced expression of *TLR3* in several cancer cell lines carrying such p53 altered alleles. The cell line RAJI, which harbors the loss-of-function mutant alleles R213Q and Y234H [34–36] for p53 targets including *TLR3* and *p21* (Supplementary Figure S4A), was treated with the reactivating drugs RITA [36] and PRIMA-1 [37] as well as with the anticancer drugs doxorubicin (DXR) and 5-fluorouracil (5FU) to induce p53.

**Figure 5:**
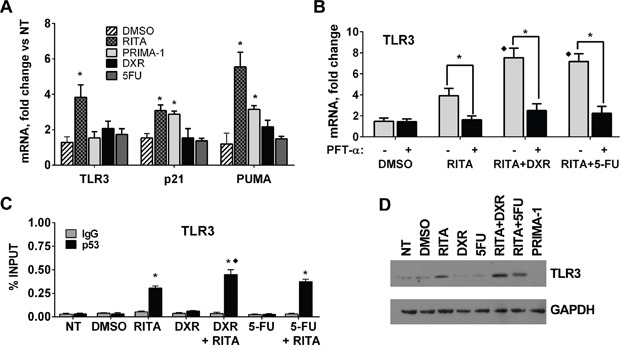
p53 reactivating molecule RITA rescues TLR3 gene expression in a p53 mutant cell line **A.** Mutant p53 RAJI cells were treated for 24h with p53 reactivating drugs RITA (1 μM) or PRIMA-1 (10 μM) or the chemotherapeutic agents DXR (0.5 μM) or 5FU (300 μM). DMSO was used as vehicle. mRNA levels of TLR3, p21 and PUMA were determined by qPCR. **B.** RAJI cells were pretreated with 10 μM p53 inhibitor PFT-α beginning 3 h before challenging cells with RITA treatment in the presence or absence of DXR or 5FU. Gene expression was evaluated 24 h later. **C.** p53 occupancy in RAJI cells assessed by ChIP-PCR showing that RITA treatment can lead to mutant p53 binding to the TLR3 promoter and that this is further enhanced by DXR and 5FU. IgG served as a negative control. **D.** Western blot of RAJI cells showing that RITA treatment alone or in combination with DNA damaging drugs increased TLR3 protein levels, while PRIMA-1 had no effect. Presented are means and standard deviations from triplicate determinations in 3 independent experiments. * denotes p<0.001 relative to NT condition. In Figure “B”, ♦ denotes p<0.001 relative to RITA treatment alone.

As shown in Figure 5A, RITA and PRIMA-1 increased the expression of the p53 targets p21 and PUMA, based on qPCR measurements at 24 after their addition to cell cultures, similar to previous reports for other p53 mutants [36, 37]. However, only RITA increased *TLR3* expression. Although DXR and 5FU can activate WT p53 and even some change-of-spectrum mutants (see Figure 4B), neither DXR nor 5FU treatment alone affected *TLR3* expression in the RAJI cells. Yet, the combination of RITA, but not PRIMA, with DXR or 5FU led to a considerable increase in *TLR3*, *p21* and *PUMA* expression (Figure 5B, Supplementary Figure S4), suggesting different mechanisms for restoring function and its potential transcriptional targets. To exclude the possibility that *TLR3* was induced through p53-independent pathways activated after p53 mutant reactivation, we incubated the cells prior to RITA treatment with the p53-specific inhibitor pifithrin-α (PFT-α) [38]. Treatment with PFT-α reduced *TLR3* as well as *P21* and *PUMA* mRNA expression to baseline levels, demonstrating the direct involvement of the p53 pathway (Figure 5B and Supplementary Figure S4) in the RITA and RITA+DXR or RITA+5FU treated cells. The increases in expression are reflected in p53 protein occupancy at the TLR3 promoter (Figure 5C and Supplementary Figure S4) and an increase in TLR3 protein (Figure 5D). RITA treatment also rescued the expression of *TLR3, p21* and *PUMA* in other cancer cells harboring hotspot p53 mutations including R175H, R273H and R280K (Supplementary Figure S5A), although in two of the cells lines RITA treatment alone resulted in massive cell death (Supplementary Figure S5B).

**Figure 6:**
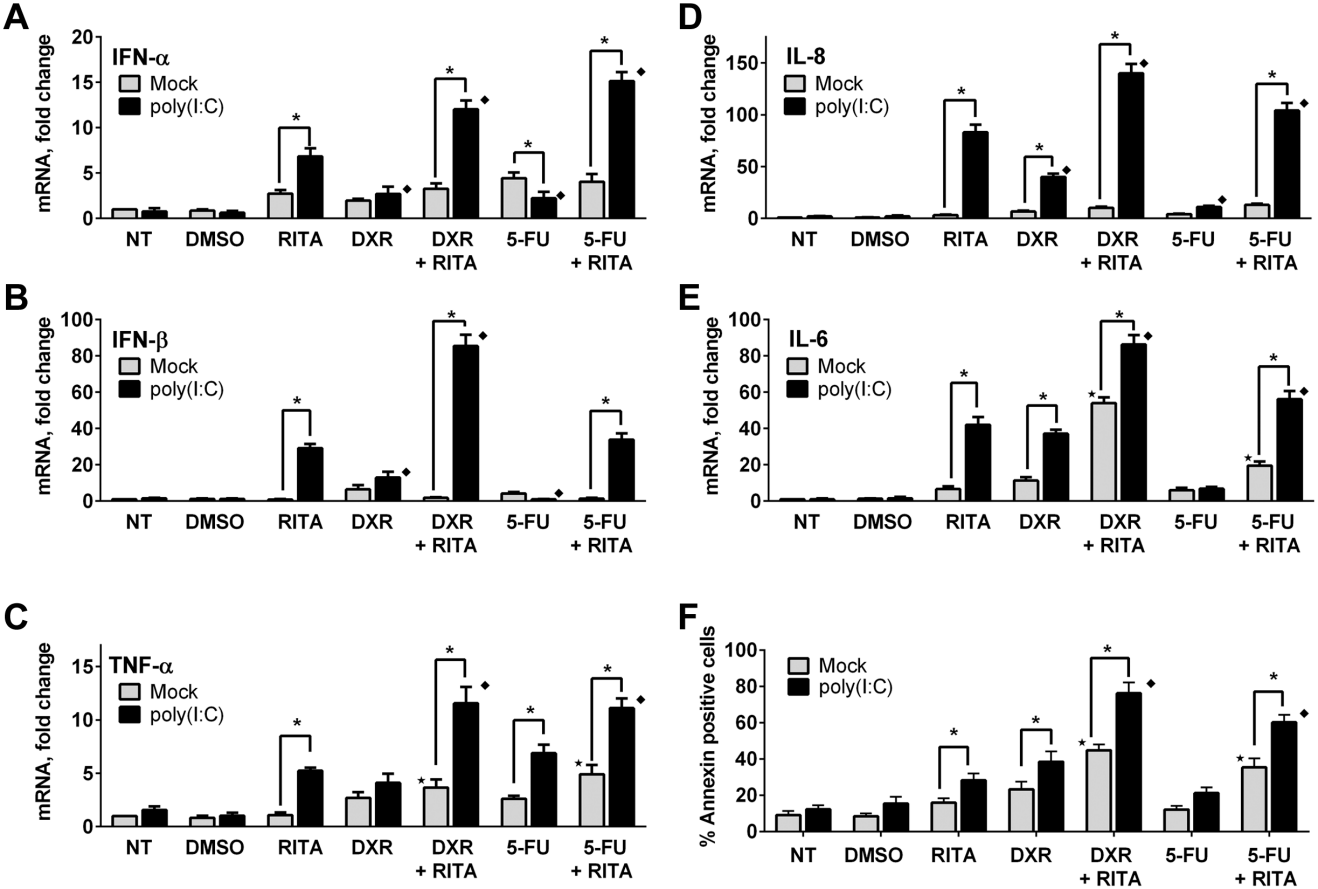
Functional reactivation of mutant p53 modulates TLR3 induced downstream signaling in RAJI cells **A-E.** Reactivation of mutant p53 in RAJI cells by RITA can increase interferon and cytokine responses to the TLR3 ligand poly(I:C). This can be increased further by DXR and to a lesser extent by 5FU. Twenty four h after drug treatment, RAJI cells were incubated for an additional 3 h with or without poly(I:C) (5 μg/mL). Gene expression was assessed by qPCR. **F.** Combination of RITA with anticancer drugs enhanced TLR3 mediated apoptosis. Apoptosis was analyzed 24 h after poly(I:C) treatment in the presence or absence of RITA (1μM), DXR (0.5 μM) or 5FU (300 μM) by Annexin-V staining as described in the Material and Methods. Presented are the median and SD of 3 independent experiments. *p<0.001 relative to mock condition. Where indicated, p<0.001 relative to RITA without (⋆) or with (♦) poly(I:C) treatment.

Since there was significant restoration of *TLR3* expression in RAJI cells after co-treatment with RITA and DNA damaging agents, we determined the effect of p53 mutant rescue by RITA on immune and apoptotic responses in RAJI cells following incubation with the TLR3 ligand poly(I:C). RITA, DXR and 5FU alone had no or just a few-fold effect on mRNA expression of *IFN-α, IFN-β, TNF-α, IL-6 and IL-8*, as shown in Figure 6A-6E. However, treatment with RITA led to a substantial increase in the downstream effect of poly(I:C) consistent with an impact of restored p53 on TLR3 expression. Both DXR and 5FU increased poly(I:C) signaling for some of the cytokines and this was generally additive with the effect of RITA. 5FU. The effects on apoptosis were somewhat similar. As shown in Figure 6F, both RITA and DXR alone could increase the level of apoptosis approximately 2-3 fold, and this was increased by the addition of poly(I:C). A further, nearly additive increase was observed with the combination of poly(I:C) and RITA+DXR, reaching approximately 80% apoptosis in 24 hr. Similar results were found with poly(I:C) and RITA+5FU. Together, these results show that functional restoration of endogenous p53 mutants can increase *TLR3* expression and synergize with DNA damaging agents to modulate TLR3 signaling responses in the presence of a cognate ligand.

## DISCUSSION

Crosstalk between tumor cells and cells of the microenvironment is crucial for regulating tumorigenesis [39]. Although the function of p53 as a barrier to cancer development has been extensively examined, recent studies have shown non-cell-autonomous effects of p53 in stimulating an anti-tumorigenic microenvironment [10]. For example, Xue et al. [40] found that p53-dependent tumor regression in a mouse liver carcinoma model was related to induction of a senescence program through the upregulation of pro-inflammatory cytokines and activation of innate immune response, characterized by the recruitment of neutrophils, macrophages and natural killer (NK) cells. This senescence-associated phenotype identified with secretion of pro-tumorigenic cytokines and chemokines can be restrained or qualitatively modified by p53 in several cell types in mouse [39, 41]. Moreover, gene expression analyses has shown that p53 directly induces numerous genes involved in triggering the recruitment of immune cells and immune surveillance in human cells [11], which can involve the TLR gene family.

Besides the canonical pathogen protection functions of TLRs, they are also expressed in many cancer epithelial cells and can affect tumor growth [18]. In addition, TLRs are activated by a wide array of endogenous molecules released from self-cells following tissue damage (known as damage-associated molecular pattern, DAMP) such as hyaluronic acid and molecules derived from necrotic and apoptotic cells including nucleic acids [42]. Many DAMPS are generated by cancer cells in response to chemotherapeutic drugs such as DXR and cyclophosphamide [43]. The interpretation of such signals by innate immune responding cells such as macrophages or dendritic cells can stimulate tumor-specific immune responses that in some settings enhance anticancer therapy. TLR antagonists as well as agonists of their negative regulators are now being developed for the treatment of cancer, allergies and viral infections and as vaccine adjuvants to enhance immune responses against tumors and infectious diseases [44, 45].

Since p53 can directly influence expression of the TLR gene family and downstream immune/inflammatory responses [24–26], understanding the relationship between p53 and its mutants with the immune system is expected to be important for addressing tumor progression and therapeutic approaches, especially since many therapies result in DNA damage and induction of available p53. Here, we showed differences in the TLR responsiveness to tumor-associated p53 mutants. In addition to loss or simple reduction, some mutants resulted in a change-in-spectrum with regards to induction of individual TLR genes. These results suggest differences in opportunities to exploit p53 mutant status in p53 mediated immune responses, especially if p53 could be reactivated.

Using TLR3 and the poly(I:C) agonist as a model, we have shown how p53 mutants can vary in their influence on immune and apoptotic signaling mediated by this receptor in response to a ligand, with some mutants retaining a strong p53 mediated enhancement of ligand responsiveness. The immune response stimulated via TLR3 has been proposed for development of anticancer therapies, and several poly(I:C) derived agonists of TLR3 are under clinical investigation [31, 46]. In addition, TLR3 has been implicated in the induction of apoptosis in response to genotoxic stresses or immune challenges. For example, Paone et al., [28] showed that poly(I:C) treatment induced TLR3 dependent apoptosis in human prostate cancer cells. Particularly relevant to the current findings was that cell lines with mutant p53 were less responsive. Moreover, Taura et al. [29] reported that anticancer drugs including 5FU, cisplatin, etoposide, and interferons could increase poly(I:C)-induced cell death in HCT116 cells that are WT p53. Based on our findings, we speculate that treatment with poly(I:C) agonists alone or in the presence of traditional chemotherapeutic agents may be more effective in patients with tumors that have TLR3-enhancing p53 mutants, such as P151H and R337H compared to A138V and G245S p53 mutants (that have no effect on TLR3 responses). Additionally, the use of poly(I:C) agonists may be best for types of cancer that have a high incidence of TLR3-enhancing p53 mutants like the R337H mutation, that has been identified in subjects with Li-Fraumeni-like syndrome with pediatric adrenal cortical carcinoma, which has a significantly increased frequency in Brazil [47]. Thus, knowing the p53 mutation status of tumor cells could be used to predict the responsiveness of cancer patients to poly(I:C) agonists in the clinic.

Based on our TLR3 results, expression profiling of other TLR gene family members in response to cancer-related p53 mutants (Figure 1) may open additional strategies for their use in therapies. We recently demonstrated that Nutlin, an activator of WT p53 functions, can greatly enhance the TLR5 downstream mediated responses to its natural ligand flagellin [26]. Among the mutants evaluated in the present study, P151H, H178Y and P190L resulted in increased TLR5 expression, whereas the M133T mutant down-regulated expression.

Since most of the commonly found p53 mutations are loss-of-function, pharmacological restoration of p53 function is an obvious therapeutic strategy against human cancers. Over the past decade, several small molecules have been identified that inactivate mutant p53 or restore WT p53 response in mutant p53 protein including PRIMA-1, MIRA-1, Elipticin, CDB3, WR1065, NSC319726, p53R3 and CP-31398, although their mechanisms of action in many cases remain to be clarified [9]. In particular, RITA was found to suppress growth of cancer cell lines carrying various p53 mutations and restored the transcriptional functions of several p53 mutant alleles including R175H, R213Q, Y234H, R248W, R273H and R280K that resulted in induction of apoptosis [36, 48]. Here, we found that RITA rescued p53-induced *TLR3* expression in RAJI cells, which carry two nonfunctional p53 mutant alleles (R213Q and Y234H). In the present study, we establish a new role for RITA. It synergizes with DNA damaging drugs to increase *TLR3* expression, enhancing its immune and apoptotic responses mediated by to its agonist poly(I:C). Without functional reactivation of the p53 altered alleles in RAJI cells, treatment with DXR or 5FU alone has no significant effect on *TLR3* expression or impact on its downstream signaling. The combination of reactivation of p53 along with enhancement of target TLR genes and exposure to appropriate TLR agonists suggests unique immune approaches to the development of anti-cancer drugs and drug combinations based on immune responses via the TLR gene family [14].

A profile of *TLR* gene expression patterns in specific tumors in response to p53 and DNA damaging agents combined with knowledge of p53 expression and mutation status in these tumors can be an important tool in cancer diagnosis and in strategies that target TLR pathway for human cancer therapy. We note that while rodents have served as excellent models systems for addressing p53 functions the lack of direct p53 responsiveness of the mouse TLR gene family precludes their use in developing human therapies based on p53 modulation of TLR responses [24]. Given the diversity of p53 mutants in human cancers, it would be useful to understand the overall matrix that dictates immune responses mediated by the family of TLR proteins within the tumor microenvironment, consequences of various agonists and the impact of specific p53 mutant or WT proteins [14] following p53 activating treatments. When combined with information about p53 status and functionality, we propose that the impact of p53 activation could be additive or even synergistic in TLR targeted cancer chemotherapy. However, manipulation of TLR signaling in the context of cancer must be done judiciously, especially with regard to the effect of TLR activation on tumor cells as well as the tumor microenvironment [49]. Choices of TLR targets would be tumor specific and depend on p53 WT or mutant status. Furthermore, downstream aspects of TLR and p53 pathways have important therapeutic implications, since many of the deleterious side effects of genotoxic chemotherapies could actually result from chemoresistance arising from p53 mutations [50]. Strategies that overcome these effects without compromising normal p53 tumor suppressor function in the rest of the body would be valuable for cancer therapy [51].

## MATERIALS AND METHODS

### Cell culture and drug treatments

SaOS2 (HTB-85), RAJI (CCL86), SKBR3 (HTB-30), SW620 (CCL227) and MDA-MB-231 (HTB-26) cells were purchased from the American Type Culture Collection (ATCC, Manassas, VA) and cultured as indicated by ATCC. HCT116 p53−/− and p53+/+ cells were a gift from Dr. Bert Vogelstein (John Hopkins University, Baltimore, MD). They were grown in McCoy's 5A supplemented with 10% FBS and 1% penicillin-streptomycin (Life Technologies, Carlsbad, CA). All cell cultures were maintained at 37°C with 5% CO2. Cells were plated 18-24h before being treated with Doxorubicin (DXR, 1μM), 5-Fluorouracil (5FU, 300μM, Sigma, St Louis, MO), RITA (1μM), or PRIMA-1MET (10μM, Cayman chemicals, Ann Arbor, MI). The TLR3 agonist poly(I:C) was from Invivogen (San Diego, CA) and was used at 5 μg/mL. Where indicated, cells were pretreated with 10 μM pifithrin-α or 20 μM pan-caspase inhibitor Z-VAD-fmk (Sigma) 3 and 2 h, respectively, prior to p53 reactivation or drug treatment.

### Plasmid constructs

WT p53 expression vector pC53-SN3 and WWP-Luc (p21/WAF1 promoter; Addgene plasmid # 16451) were a gift from B. Vogelstein. All of the p53 point-mutant constructs described in the text were derived from pC53-SN3 vector and prepared using the QuikChange II XL site-directed mutagenesis kit (Stratagene, La Jolla, CA) according to the manufacturer's instructions. The TLR3 promoter:luc reporter plasmid was constructed in pGL3 Basic:luciferase vector (Promega, Madison, WI) as described in reference [23]. Sequences of all the constructs developed for this study were confirmed by DNA sequencing.

### Transfections and luciferase assays

Transient transfections with p53 mutants were carried out using FuGENE 6 reagent (Promega), according to the manufacturer's instructions. Briefly, cells were plated in 6-well plates and transfected in triplicate with 1μg of p53 expression constructs. After 24 h, cells were harvested. For luciferase assays, p53−/− HCT116 cells seeded into 12-well plates were transfected with 0.25 μg of the luciferase reporter vector together with 50ng of p53 expression vector; 25 ng of pRL-TK Renilla luciferase plasmid (Promega) was used as a transfection efficiency control. Luciferase activities were measured 48 h post-transfection with a Synergy2 Multi-mode Microplate reader (Bio-Tek Instruments Inc., Winooski, VT, using the dual-luciferase assay system (Promega), according to the manufacturer's protocol. Relative luciferase activity for each construct was defined as the mean value of the firefly luciferase/Renilla luciferase ratios obtained from three independent experiments, each performed in triplicate.

### Western blotting

Whole and nuclear protein fractions were prepared using RIPA lysis buffer and NE-PER extraction kit, respectively (ThermoScientifc, Cincinnati, OH), following manufacturer instructions. Equal amounts of whole cell or nuclear protein lysates, previously quantified using BCA protein assay kit (Thermo Scientific), were processed for western blotting as described [11]. The following are the antibodies used in this study: p53 (DO-1); GAPDH (6C5) and tubulin (Ab:H-90) used for loading controls (Santa Cruz Biotechnology, Santa Cruz, CA); TLR3 (3643) antibody from ProSci (Poway, CA); and p21 (SX118, BD, Biosciences, San Jose CA). The membranes were probed with appropriate primary antibodies overnight at 4°C or for 2 h at room temperature followed by incubation with the appropriate HRP-linked secondary antibodies (Santa Cruz Biotechnology). Proteins were visualized using enhanced chemiluminescence reagents (Pico Reagent, Thermo Scientific).

### Real time PCR

Total RNA was extracted from cells using the RNeasy Mini Kit in the presence of DNAse (QIAGEN, Valencia, CA). Complementary DNAs (cDNA) were generated from 1 μg of purified RNA using TaqMan reverse transcription reagents (Applied Biosystems, Foster City, CA); then, TaqMan RT-PCR was performed with the HT7900 system (Applied Biosystems) using *TLRs*, *p21* and *B2M* pre-validated primers, as described in [24–26].

### Cytokine measurement

Secreted IL-6 from treated cells was quantified using the IL-6 Human ELISA Kit from eBioscience (San Diego, CA) following the manufacturer's protocol.

### Chromatin immunoprecipitation assay (ChIP)

ChIP assays were performed as described [11] in triplicate. Briefly, after treatment, the cellular material was cross-linked with methanol free 1% formaldehyde (Sigma). Then, cell lysates were sonicated using conditions that yield 200-500 bp DNA fragments using a Bioruptor XL (Diagenode, Denville, NJ). DNA-protein complexes were immunoprecipitated with 1 μg of DO-1 p53-specific monoclonal antibody per condition. Mouse Ig (Santa Cruz Biotechnology) was used as a negative control. qPCR was performed on immunoprecipitated chromatin to determine p53 enrichment occupancy on TLR3 and p21 promoter regions. Amplification of *GADPH* promoter region was used as a negative control. ChIP primers for *TLR3, p21* and *GADPH* were previously described [23–24]. qPCR and melting curve analysis was performed using the SYBR^®^ Green (Invitrogen) dye detection method on the ABI PRISM 7900 HT Sequence Detection System under default conditions. Enrichment in the ChIP samples was calculated as a fraction of the Input (%).

### Apoptosis detection

Briefly, after the indicated treatment, both floating and non-floating cells were collected and washed twice in PBS. The level of apoptosis was measured by flow cytometry using the Annexin V/PI assay (BD, Biosciences) according to the manufacturer's protocol using BD LSRII (BD, Biosciences) equipment. Data were collected on 10,000 cells. Apoptotic fractions were determined where cells positive for Annexin V staining but not propidium iodide were counted.

### Cellular proliferation assay

Cells were seeded into 96-well plates at 2,000 cells/well, in 100 μL of medium and incubated for 24 h. The next day, cells were exposed to the indicated drugs for the indicated times. Following treatments, the CellTiter 96 One Solution assay (Promega) was used to measure cell proliferation status, per manufacturer's instructions. Optical densities were measured using a Synergy2 Multi-mode Microplate reader (Bio-Tek Instruments). Triplicate wells were assayed for each condition.

### Statistics

Data were graphed and analyzed with Graphpad Prism6 software. The data presented correspond to the mean ± SD of at least three experiments where each was done in triplicate. Statistically significant differences were identified using two way ANOVA. p-values < 0.001 were considered statistically significant.

## SUPPLEMENTARY FIGURES AND TABLES





## References

[1] Vousden KH, Prives C (2009). Blinded by the Light: The Growing Complexity of p53. Cell.

[2] Kandoth C, McLellan MD, Vandin F, Ye K, Niu B, Lu C, Xie M, Zhang Q, McMichael JF, Wyczalkowski MA, Leiserson MDM, Miller CA, Welch JS, Walter MJ, Wendl MC, Ley TJ (2013). Mutational landscape and significance across 12 major cancer types. Nature.

[3] Martincorena I, Campbell PJ (2015). Somatic mutation in cancer and normal cells. Science.

[4] Resnick MA, Inga A (2003). Functional mutants of the sequence-specific transcription factor p53 and implications for master genes of diversity. Proceedings of the National Academy of Sciences of the United States of America.

[5] Kato S, Han SY, Liu W, Otsuka K, Shibata H, Kanamaru R, Ishioka C (2003). Understanding the function-structure and function-mutation relationships of p53 tumor suppressor protein by high-resolution missense mutation analysis. Proceedings of the National Academy of Sciences of the United States of America.

[6] Menendez D, Inga A, Resnick MA (2006). The biological impact of the human master regulator p53 can be altered by mutations that change the spectrum and expression of its target genes. Molecular and cellular biology.

[7] Jordan JJ, Inga A, Conway K, Edmiston S, Carey LA, Wu L, Resnick MA (2010). Altered-function p53 missense mutations identified in breast cancers can have subtle effects on transactivation. Molecular cancer research.

[8] Monti P, Ciribilli Y, Jordan J, Menichini P, Umbach DM, Resnick MA, Luzzatto L, Inga A, Fronza G (2007). Transcriptional functionality of germ line p53 mutants influences cancer phenotype. Clinical cancer research.

[9] Zawacka-Pankau J, Selivanova G (2015). Pharmacological reactivation of p53 as a strategy to treat cancer. J Intern Med.

[10] Smeenk L, van Heeringen SJ, Koeppel M, van Driel MA, Bartels SJ, Akkers RC, Denissov S, Stunnenberg HG, Lohrum M (2008). Characterization of genome-wide p53-binding sites upon stress response. Nucleic acids research.

[11] Menendez D, Nguyen TA, Freudenberg JM, Mathew VJ, Anderson CW, Jothi R, Resnick MA (2013). Diverse stresses dramatically alter genome-wide p53 binding and transactivation landscape in human cancer cells. Nucleic acids research.

[12] Nikulenkov F, Spinnler C, Li H, Tonelli C, Shi Y, Turunen M, Kivioja T, Ignatiev I, Kel A, Taipale J, Selivanova G (2012). Insights into p53 transcriptional function via genome-wide chromatin occupancy and gene expression analysis. Cell death and differentiation.

[13] Bieging KT, Mello SS, Attardi LD (2014). Unravelling mechanisms of p53-mediated tumour suppression. Nature reviews Cancer.

[14] Menendez D, Shatz M, Resnick MA (2013). Interactions between the tumor suppressor p53 and immune responses. Current opinion in oncology.

[15] Lowe JM, Shatz M, Resnick M.A, Menendez D (2013). Modulation of immune responses by the tumor suppressor p53. BioDiscovery.

[16] Martins CP, Brown-Swigart L, Evan GI (2006). Modeling the therapeutic efficacy of p53 restoration in tumors. Cell.

[17] Ventura A, Kirsch DG, McLaughlin ME, Tuveson DA, Grimm J, Lintault L, Newman J, Reczek EE, Weissleder R, Jacks T (2007). Restoration of p53 function leads to tumour regression in vivo. Nature.

[18] Rakoff-Nahoum S, Medzhitov R (2009). Toll-like receptors and cancer. Nature reviews Cancer.

[19] Kawai T, Akira S (2007). Signaling to NF-kappaB by Toll-like receptors. Trends in molecular medicine.

[20] Iwasaki A, Medzhitov R (2015). Control of adaptive immunity by the innate immune system. Nature immunology.

[21] Galluzzi L, Vacchelli E, Eggermont A, Fridman WH, Galon J, Sautes-Fridman C, Tartour E, Zitvogel L, Kroemer G (2012). Trial Watch: Experimental Toll-like receptor agonists for cancer therapy. Oncoimmunology.

[22] Butt AQ, Mills KH (2014). Immunosuppressive networks and checkpoints controlling antitumor immunity and their blockade in the development of cancer immunotherapeutics and vaccines. Oncogene.

[23] Taura M, Eguma A, Suico MA, Shuto T, Koga T, Komatsu K, Komune T, Sato T, Saya H, Li JD, Kai H (2008). p53 regulates Toll-like receptor 3 expression and function in human epithelial cell lines. Molecular and cellular biology.

[24] Menendez D, Shatz M, Azzam K, Garantziotis S, Fessler MB, Resnick MA (2011). The Toll-like receptor gene family is integrated into human DNA damage and p53 networks. PLoS genetics.

[25] Shatz M, Menendez D, Resnick MA (2012). The human TLR innate immune gene family is differentially influenced by DNA stress and p53 status in cancer cells. Cancer research.

[26] Shatz M, Shats I, Menendez D, Resnick MA (2015). p53 amplifies Toll-like receptor 5 response in human primary and cancer cells through interaction with multiple signal transduction pathways. Oncotarget.

[27] Salaun B, Coste I, Rissoan MC, Lebecque SJ, Renno T (2006). TLR3 can directly trigger apoptosis in human cancer cells. Journal of immunology.

[28] Paone A, Starace D, Galli R, Padula F, De Cesaris P, Filippini A, Ziparo E, Riccioli A (2008). Toll-like receptor 3 triggers apoptosis of human prostate cancer cells through a PKC-alpha-dependent mechanism. Carcinogenesis.

[29] Taura M, Fukuda R, Suico MA, Eguma A, Koga T, Shuto T, Sato T, Morino-Koga S, Kai H (2010). TLR3 induction by anticancer drugs potentiates poly I:C-induced tumor cell apoptosis. Cancer Sci.

[30] Gambara G, Desideri M, Stoppacciaro A, Padula F, De Cesaris P, Starace D, Tubaro A, Del Bufalo D, Filippini A, Ziparo E, Riccioli A (2015). TLR3 engagement induces IRF-3-dependent apoptosis in androgen-sensitive prostate cancer cells and inhibits tumour growth in vivo. J Cell Mol Med.

[31] Glavan TM, Pavelic J (2014). The exploitation of Toll-like receptor 3 signaling in cancer therapy. Current pharmaceutical design.

[32] Muller PA, Vousden KH (2013). p53 mutations in cancer. Nature cell biology.

[33] Alexopoulou L, Holt AC, Medzhitov R, Flavell RA (2001). Recognition of double-stranded RNA and activation of NF-kappaB by Toll-like receptor 3. Nature.

[34] Chow VT, Quek HH, Tock EP (1993). Alternative splicing of the p53 tumor suppressor gene in the Molt-4 T-lymphoblastic leukemia cell line. Cancer Lett.

[35] Farrell PJ, Allan GJ, Shanahan F, Vousden KH, Crook T (1991). p53 is frequently mutated in Burkitt's lymphoma cell lines. EMBO J.

[36] Zhao CY, Grinkevich VV, Nikulenkov F, Bao W, Selivanova G (2010). Rescue of the apoptotic-inducing function of mutant p53 by small molecule RITA. Cell cycle.

[37] Bykov VJ, Issaeva N, Shilov A, Hultcrantz M, Pugacheva E, Chumakov P, Bergman J, Wiman KG, Selivanova G (2002). Restoration of the tumor suppressor function to mutant p53 by a low-molecular-weight compound. Nature medicine.

[38] Komarov PG, Komarova EA, Kondratov RV, Christov-Tselkov K, Coon JS, Chernov MV, Gudkov AV (1999). A chemical inhibitor of p53 that protects mice from the side effects of cancer therapy. Science.

[39] Hanahan D, Coussens LM (2012). Accessories to the crime: functions of cells recruited to the tumor microenvironment. Cancer cell.

[40] Xue W, Zender L, Miething C, Dickins RA, Hernando E, Krizhanovsky V, Cordon-Cardo C, Lowe SW (2007). Senescence and tumour clearance is triggered by p53 restoration in murine liver carcinomas. Nature.

[41] Lujambio A, Akkari L, Simon J, Grace D, Tschaharganeh DF, Bolden JE, Zhao Z, Thapar V, Joyce JA, Krizhanovsky V, Lowe SW (2013). Non-cell-autonomous tumor suppression by p53. Cell.

[42] Chen GY, Nunez G (2010). Sterile inflammation: sensing and reacting to damage. Nature reviews Immunology.

[43] Zitvogel L, Kepp O, Kroemer G (2010). Decoding Cell Death Signals in Inflammation and Immunity. Cell.

[44] Kanzler H, Barrat FJ, Hessel EM, Coffman RL (2007). Therapeutic targeting of innate immunity with Toll-like receptor agonists and antagonists. Nature medicine.

[45] Hedayat M, Takeda K, Rezaei N (2012). Prophylactic and therapeutic implications of toll-like receptor ligands. Medicinal research reviews.

[46] Hussein WM, Liu TY, Skwarczynski M, Toth I (2014). Toll-like receptor agonists: a patent review (2011 - 2013). Expert opinion on therapeutic patents.

[47] Garritano S, Gemignani F, Palmero EI, Olivier M, Martel-Planche G, Le Calvez-Kelm F, Brugieres L, Vargas FR, Brentani RR, Ashton-Prolla P, Landi S, Tavtigian SV, Hainaut P, Achatz MI (2010). Detailed haplotype analysis at the TP53 locus in p.R337H mutation carriers in the population of Southern Brazil: evidence for a founder effect. Human mutation.

[48] Hiraki M, Hwang SY, Cao S, Ramadhar TR, Byun S, Yoon KW, Lee JH, Chu K, Gurkar AU, Kolev V, Zhang J, Namba T, Murphy ME, Newman DJ, Mandinova A, Clardy J (2015). Small-Molecule Reactivation of Mutant p53 to Wild-Type-like p53 through the p53-Hsp40 Regulatory Axis. Chemistry & biology.

[49] Goutagny N, Estornes Y, Hasan U, Lebecque S, Caux C (2012). Targeting pattern recognition receptors in cancer immunotherapy. Target Oncol.

[50] Selivanova G, Wiman KG (2007). Reactivation of mutant p53: molecular mechanisms and therapeutic potential. Oncogene.

[51] Gudkov AV, Komarova EA (2003). The role of p53 in determining sensitivity to radiotherapy. Nature reviews Cancer.

