# Supplementary material for: Ligand dependent restoration of human TLR3 signaling and death in p53 mutant cells
Source: Oncotarget. 2016 Aug 11;7(38):61630–42. doi: 10.18632/oncotarget.11210 (PMC5308678; doi:10.18632/oncotarget.11210)
Supplement: Supplementary file 1 [file oncotarget-07-61630-s001.pdf]

Ligand dependent restoration of human TLR3 signaling and death in p53 mutant cells

SUPPLEMENTARY FIGURES AND TABLES

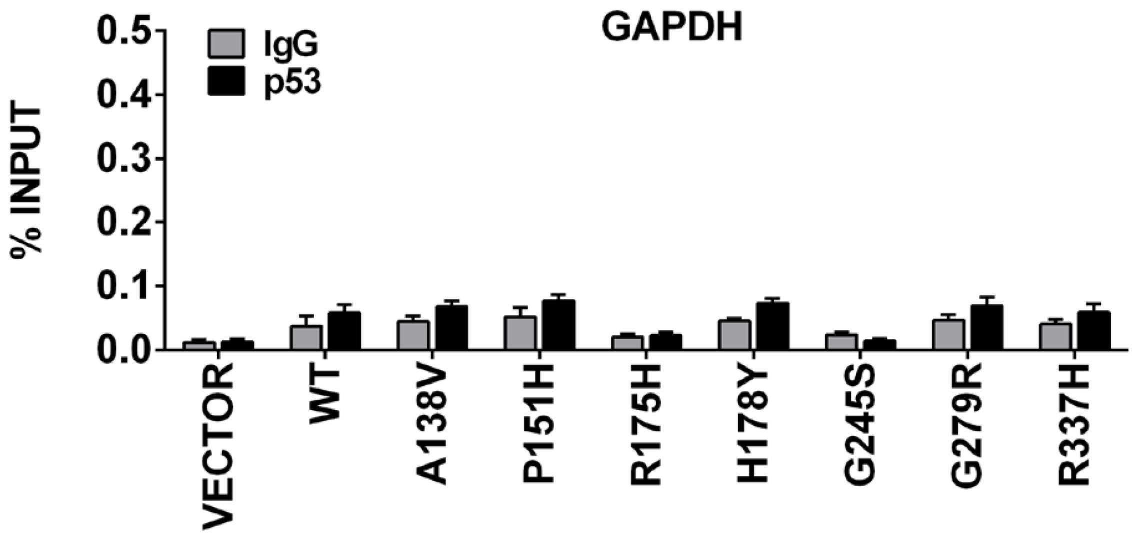

Supplementary Figure S1: Lack of p53 occupancy in the GAPDH promoter in HCT116 p53-/- cells transfected with WT or mutant p53 expression vectors.

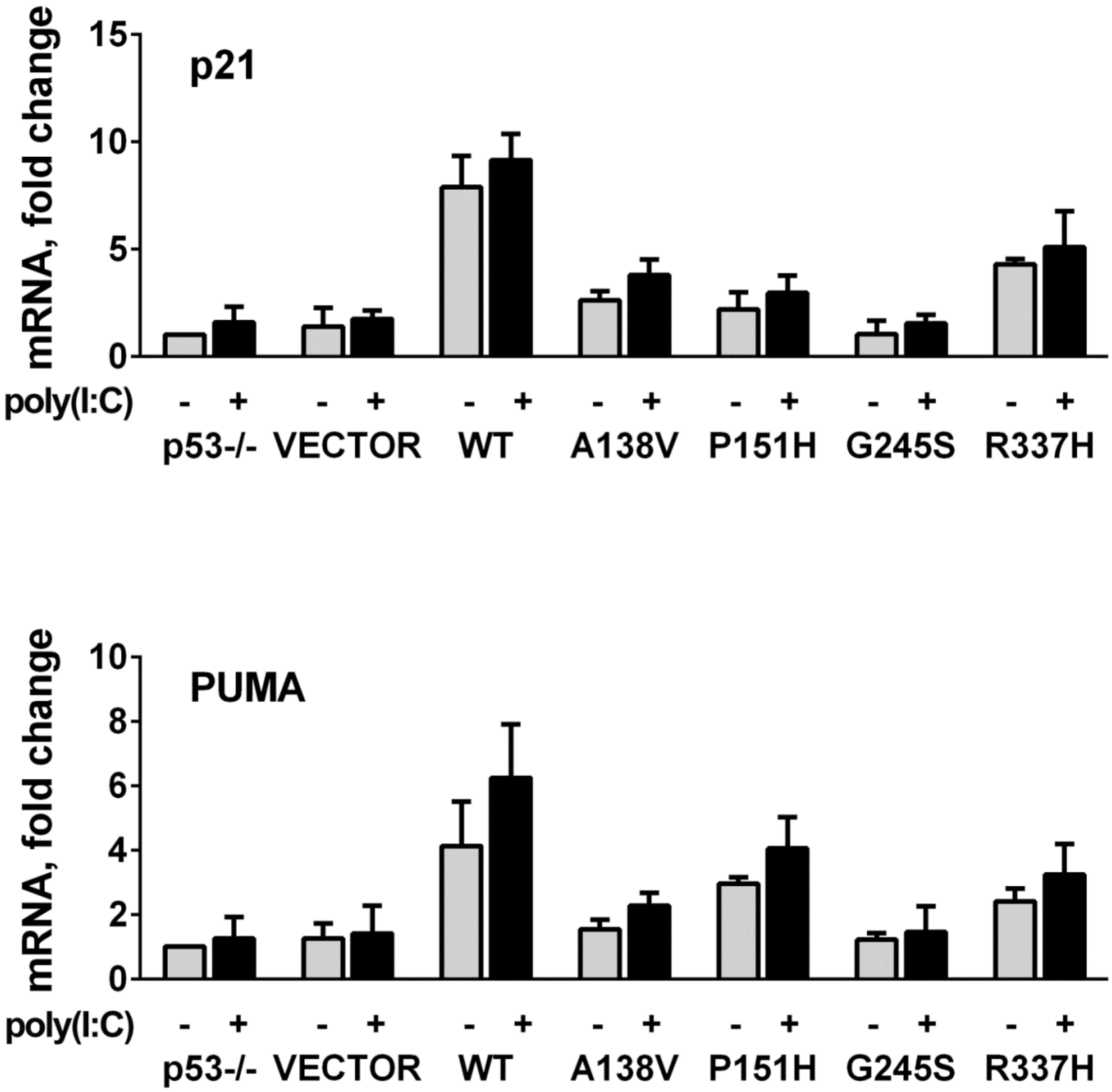

Supplementary Figure S2: Effect of poly(I:C) treatment on *p21* and *PUMA* expression in HCT116 p53-/- cells transfected with plasmids containing WT or mutant p53.

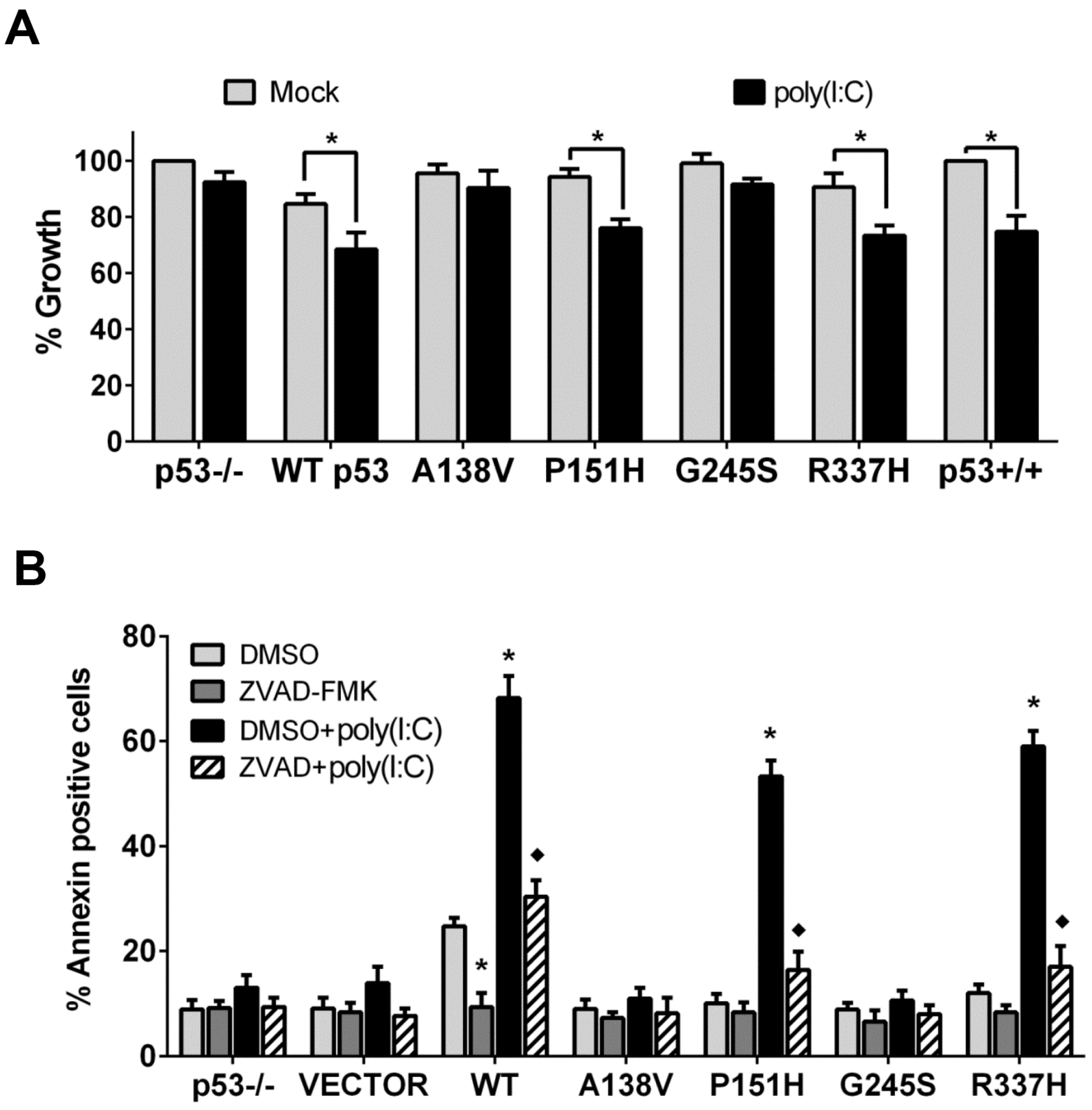

Supplementary Figure S3: p53 mutants can enhance poly (I:C) induced TLR3 dependent cell death. A. MTT based cell proliferation assay in HCT116 p53-/- cells transfected with WT and mutant p53 plasmids. After 24h, cells were treated with TLR3 ligand poly(I:C) (5µg/ mL). MTT assay was run 24h later. **B.** Pan-caspase inhibitor z-VAD-fmk inhibits poly(I:C) induced apoptosis as analyzed by Annexin V/PI assay. * p<0.001 relative to DMSO; ♦ p<0.001 relative to DMSO plus poly(I:C).

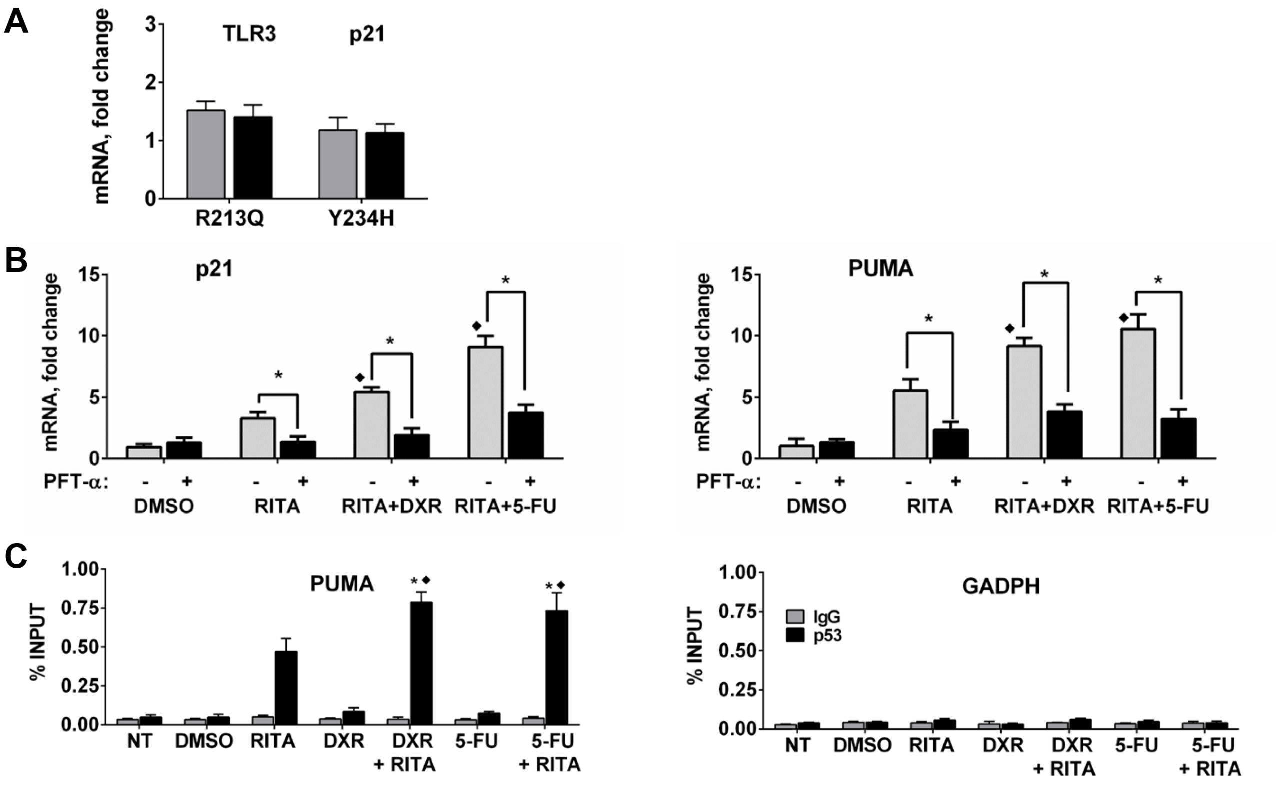

Supplementary Figure S4: A. R213Q and Y234H p53 mutants can not induce *TLR3* and *p21* expression determined by qPCR, when expressed in p53 null HCT116 cells. **B.** Restoration of mutant p53 transcriptional function by RITA in RAJI cells increases along with and DXR and 5-FU to modulate the expression of *p21* and *PUMA* (B) while pretreatment of cells with the p53 inhibitor PFT-α (10μM) abolishes RITA effect. **C.** RITA treatment rescued p53 mutant binding on the PUMA promoter, as determined by ChiP-PCR. GADPH control for binding. * denotes p<0.001 and "♦" denotes <0.001 relative to RITA treatment alone.

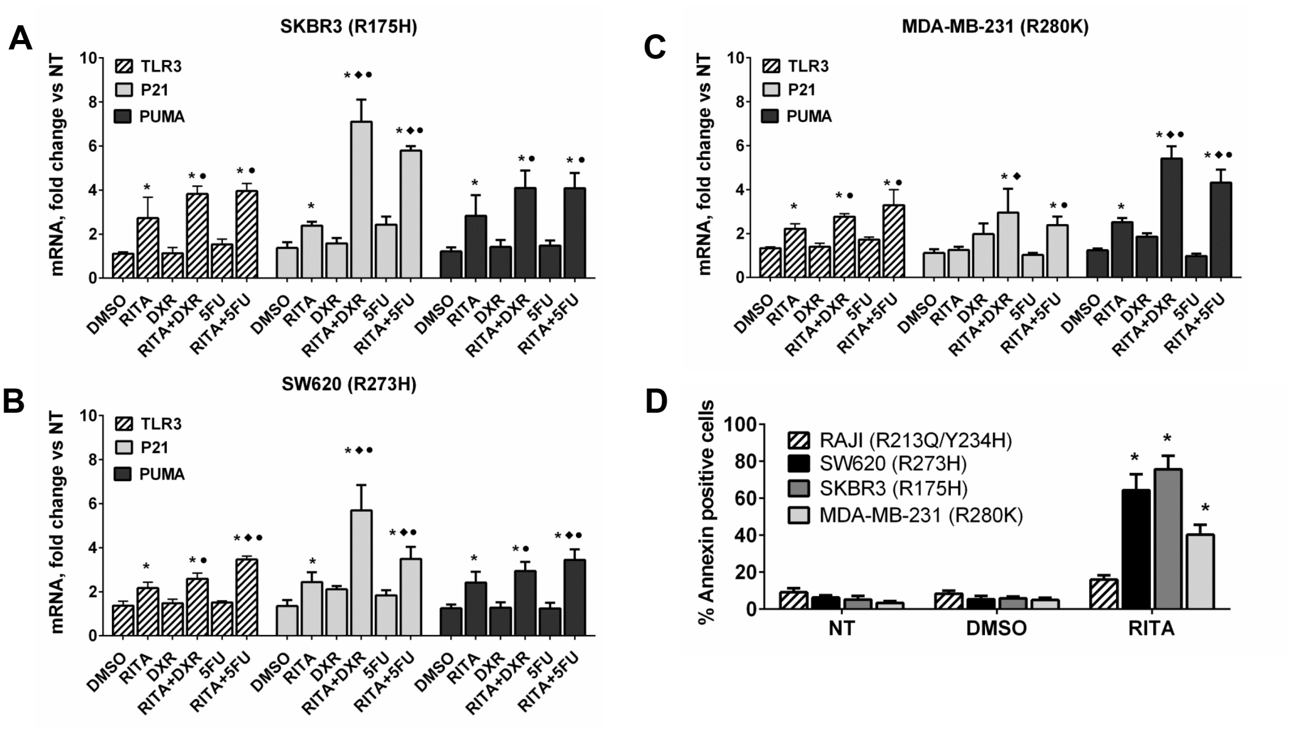

Supplementary Figure S5: A-C. p53 reactivating molecule RITA rescues *TLR3*, *p21* and *PUMA* gene expression in a p53 mutant cell lines harboring different p53 loss of function alleles. Cell lines were treated for 24h with RITA (1 µM) in presence or absence of the chemotherapeutic agents DXR (0.5 µM) or 5FU (300 µM). DMSO was used as vehicle. mRNA levels of *TLR3*, *p21* and *PUMA* were determined by qPCR. **D.** RITA induces apoptosis in p53 mutant cell lines Apoptosis was analyzed 48 h after RITA (1µM) treatment by Annexin-V staining. Presented are the average and SD of 3 independent experiments. *p<0.001 relative to No treatment (NT) condition. "♦" and "●" denote <0.001 relative to RITA treatment alone and chemoterapeutic drug alone respectively.

Supplementary Table S1: Functional description and tumor appearance frequencies for p53 mutants used in this study

MUTANT	FUNCTIONAL STATUS		IARC DATABASE R18 April 2016[c]	
	Resnick lab[a]	Transactivation class[b]	Somatic †	Germinal †
S121F	Functional (ST)	functional	2	0
V122A	Altered	non-functional	0	0
T125R	Altered	non-functional	2	1
M133T	Altered	non-functional	7	25
A138V	Functional	partially functional	55	4
P151H	Altered	non-functional	42	0
R175H	Non-functional	non-functional	1216	75
H178Y	Functional	partially functional	13	0
P190L	Altered	partially functional	52	3
Y220C	Altered	non-functional	402	38
M237I	Altered	non-functional	196	2
G245D	Non-functional	non-functional	162	8
G245S	Non-functional	non-functional	456	40
V272L	Functional	non-functional	52	1
R273C	Non-functional	non-functional	707	35
R273H	Non-functional	non-functional	858	63
C277W	Altered	non-functional	6	0
G279E	Non-functional	non-functional	53	0
G279R	Altered	non-functional	9	1
R280K	Non-functional	non-functional	75	1
N288K	Functional (ST)	functional	2	0
R337C	Altered	non-functional	20	8
R337H	Altered	partially functional	4	127
L344P	Non-functional	non-functional	2	9
N345S	Altered	functional	0	0

[a] Assessment of transactivation capacities in yeast assays by Resnick et al., [4] and Jordan et al,. [7]
[b] Assessment of transactivation capacities in yeast assays by Kato et al., [5]
[c] Petitjean A, Mathe E, Kato S, Ishioka C, Tavtigian SV, Hainaut P, Olivier M. Impact of mutant p53 functional properties on TP53 mutation patterns and tumor phenotype: lessons from recent developments in the IARC TP53 database. Hum Mutat. 2007; 28:622-629.
ST= Supertransactivation
† Number of reported allele-specific mutations in the R18 release of the IARC p53 mutation database: over 29000 somatic mutations, and 891 germline mutations
IARC TP53 DATABASE http://p53.iarc.fr

Supplementary Table S2: mRNA fold-change values for *TLR* gene expression profiles in HCT116 p53-/- cells transfected with p53 mutants. mRNA fold-change values for *TLR* gene expression profiles in SaOS2 cells transfected with p53 mutants.

See Supplementary File 1
